# The SCF Complex Is Essential to Maintain Genome and Chromosome Stability

**DOI:** 10.3390/ijms22168544

**Published:** 2021-08-09

**Authors:** Laura L. Thompson, Kailee A. Rutherford, Chloe C. Lepage, Kirk J. McManus

**Affiliations:** 1 CancerCare Manitoba Research Institute, CancerCare Manitoba, Winnipeg, MB R3E 0V9, Canada; Thompson.Laura1@mayo.edu (L.L.T.); rutherf6@myumanitoba.ca (K.A.R.); lepagec@myumanitoba.ca (C.C.L.); 2 Department of Biochemistry & Medical Genetics, University of Manitoba, Winnipeg, MB R3E 0J9, Canada

**Keywords:** cancer, cell cycle, chromosome instability, chromosome stability, F-box protein, genome instability, SCF complex, therapeutic targeting, transcription, ubiquitin–proteasome system

## Abstract

The SKP1, CUL1, F-box protein (SCF) complex encompasses a group of 69 SCF E3 ubiquitin ligase complexes that primarily modify protein substrates with poly-ubiquitin chains to target them for proteasomal degradation. These SCF complexes are distinguishable by variable F-box proteins, which determine substrate specificity. Although the function(s) of each individual SCF complex remain largely unknown, those that have been characterized regulate a wide array of cellular processes, including gene transcription and the cell cycle. In this regard, the SCF complex regulates transcription factors that modulate cell signaling and ensures timely degradation of primary cell cycle regulators for accurate replication and segregation of genetic material. SCF complex members are aberrantly expressed in a myriad of cancer types, with altered expression or function of the invariable core SCF components expected to have a greater impact on cancer pathogenesis than that of the F-box proteins. Accordingly, this review describes the normal roles that various SCF complexes have in maintaining genome stability before discussing the impact that aberrant SCF complex expression and/or function have on cancer pathogenesis. Further characterization of the SCF complex functions is essential to identify and develop therapeutic approaches to exploit aberrant SCF complex expression and function.

## 1. Introduction

The SKP1, CUL1, F-box protein (SCF) complex is an E3 ubiquitin ligase that covalently attaches mono- or poly-ubiquitin chains onto protein targets. These distinctive protein modifications invoke a plethora of spatial and temporal outcomes, including altering protein localization, regulating protein activity, or targeting a protein for proteolytic degradation (reviewed in [1,2]). While each of these outcomes ultimately impact protein function, arguably the best studied relationship is that between protein poly-ubiquitination and the ubiquitin–proteosome system (UPS). In this regard, the SCF complex poly-ubiquitinates protein substrates targeting them for proteolytic degradation by the 26S proteasome. Under normal conditions, the temporal degradation of specific proteins is critical to regulate many biological processes that are essential to maintain genome stability, including the regulation of various signal transduction cascades, cell cycle progression, DNA repair, and apoptosis. As a result, it is not difficult to envision how genomic alterations affecting the genes encoding the SCF complex will interfere with these key biological processes and promote genome instability to effectively contribute to cancer development and progression.

The focus of this review is to present evidence supporting the possibility that aberrant SCF complex expression underlies both tumor suppressor- and oncogene-like activities and has a potential impact on cancer development and progression. We first provide a brief background on the UPS and detail its central role in regulating protein abundance. We then describe the SCF complex, with a particular focus on the core and variable members that comprise the complex. We subsequently highlight several key biological processes that are regulated by the SCF complex and have strong pathogenic implications for cancer, including the regulation of transcription, downstream cancer-associated signaling pathways, and cell cycle, with our companion review article [3] focused on the DNA damage response, apoptosis, and centrosome biology. Finally, we end with a discussion of the most frequent types of genetic alterations involving the invariable core SCF complex members before presenting some future therapeutic strategies aimed at exploiting aberrant SCF complex expression and function, and the downstream impacts of aberrant protein turnover.

## 2. Ubiquitin-Dependent Proteasomal Degradation

**Figure 1:**
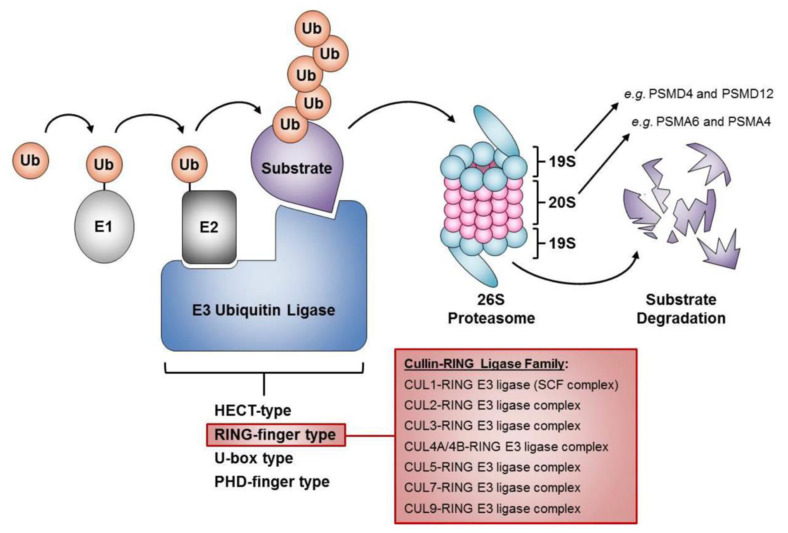
The Components of the ubiquitin–proteasome system. Substrates intended for degradation by the 26S proteasome are poly-ubiquitinated through the concerted activities of an E1 ubiquitin-activating enzyme (E1), an E2 ubiquitin-conjugating enzyme (E2), and an E3 ubiquitin-protein ligase (E3). The four major E3 ubiquitin ligases families are listed and include the HECT, RING-finger, U-box, and PHD-finger families. Additionally, the seven RING-finger type Cullin-based ligases are listed (red box), which includes the prototypic CUL1-based SCF complex. Ub, Ubiquitin; PSMD4, Proteasome 26S Subunit Non-ATPase 4; PSMD12, Proteasome 26S Subunit Non-ATPase 12; PSMA6, Proteasome Subunit Alpha 6; and PSMA4, Proteasome Subunit Alpha 4.

The UPS is a major mechanism of targeted intracellular protein degradation that is essential for the spatial and temporal regulation of protein abundance (reviewed in [4]). The UPS consists of a large network of proteins and/or enzymes that collectively act to regulate the abundance of protein targets through two successive steps (Figure 1). First, a ubiquitin moiety is covalently attached to a lysine residue contained within a protein target and is subsequently modified through the repeated addition of ubiquitin moieties to ultimately produce a poly-ubiquitin chain. The poly-ubiquitinated protein is subsequently transported to the 26S proteasome where it undergoes proteolytic degradation [4]. Briefly, the 26S proteasome is a 2.5 megadalton macromolecular structure containing a cylindrical 20S catalytic subunit harboring peptidase activity and a ring-shaped 19S regulatory subunit consisting of structural components, ubiquitin receptors, and adenosine triphosphatases (ATPases) that bind, denature, and translocate protein targets into the 20S proteolytic core [5].

Substrate poly-ubiquitination provides a high degree of specificity for the UPS and is performed by the sequential actions of three enzymes (Figure 1): (1) an E1 ubiquitin-activating enzyme; (2) an E2 ubiquitin-conjugating enzyme; and (3) an E3 ubiquitin ligase [6]. These three enzymes are responsible for the adenosine triphosphate (ATP)-dependent ubiquitin activation by the E1, the conjugation of ubiquitin to the E2, and the transfer of ubiquitin to the protein target by the E3 to ultimately create a poly-ubiquitin chain linked through a series of lysine 48 (K48) to glycine 76 (G76) isopeptide bonds [6]. As it is the E3 enzyme that dictates substrate specificity for the UPS, a large number of E3 ubiquitin ligases (~650) exist within the human genome (reviewed in [7]). The E3 ubiquitin ligases are divided into four main groups based on the E2-binding structural motif that they harbor, including the Homologous to the E6-AP Carboxyl Terminus (HECT), the U-box, the plant homeo domain (PHD)-finger, and the really interesting new gene (RING)-finger type (Figure 1). The RING-finger ligases are further classified into subfamilies, with the largest being the Cullin-based subfamily [8], of which the SCF complex is considered the prototypical Cullin-based, RING-finger type E3 enzyme.

## 3. The SCF Complex

**Figure 2:**
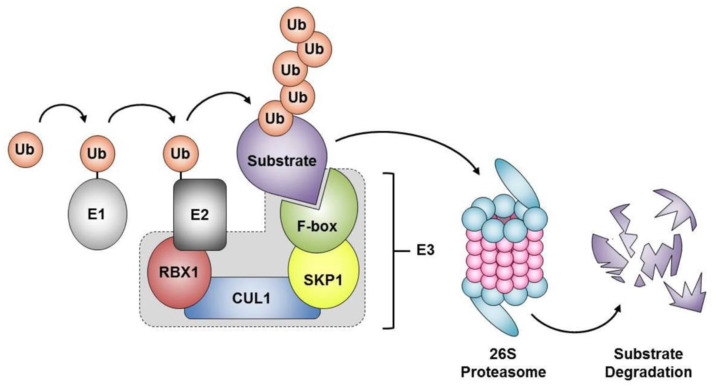
The SCF complex orchestrates proteolytic degradation by the 26S proteasome. Schematic of the SCF complex consisting of four components, including invariant core members (SKP1, RBX1 and CUL1) and one of the 69 variable F-box proteins. The F-box proteins facilitate the transfer of ubiquitin from the E2-conjugating enzymes onto protein substrates, where poly-ubiquitination often denotes those targeted for proteolytic degradation via the 26S proteasome. E1, E1 ubiquitin-activating enzyme; E2, E2 ubiquitin-conjugating enzyme; E3, E3 ubiquitin-protein ligase; Ub, Ubiquitin.

The SCF complex is composed of three invariable core components, namely RING-box 1 (RBX1), a RING-finger protein that recruits the E2 ubiquitin-conjugating enzyme, cullin 1 (CUL1), the scaffolding protein, and the S-phase kinase-associated protein 1 (SKP1), an invariable adaptor component that bridges the core SCF complex with a variable F-box protein and its corresponding protein target (Figure 2) [9]. Accordingly, the target specificity of the SCF complex is imparted by the F-box proteins, each of which recognizes and binds a distinct set of substrates. There are a total of 69 F-box proteins in humans, which are organized into three families according to their substrate recognition domains: (1) FBXW harboring WD40 repeats (e.g., FBXW7); (2) FBXL with leucine-rich repeats (e.g., FBXL1, better known as the S-phase kinase associated protein 2 [SKP2]); and (3) FBXO containing other domains (e.g., FBXO28) [8]. To regulate the abundance of specific protein targets, each F-box protein recruits one of its substrates (often phospho-activated) [10] to the core SCF complex to facilitate poly-ubiquitination and subsequent degradation by the 26S proteasome (Figure 2) [11]. The presence of 69 different F-box genes implies that there are up to 69 unique SCF complexes, each of which regulates a myriad of protein targets [8]. Furthermore, SCF complex members, such as SKP1 and RBX1, have been shown to interact with non-prototypical binding partners to form additional E3 ubiquitin ligase complexes and regulate additional protein targets. For example, SKP1, RBX1, and FBXW8 can interact with CUL7 [12] to form an alternative Cullin-based RING-finger type E3 enzyme, which regulates a distinct subset of protein targets from those normally regulated by SCF^FBXW8^.

Unfortunately, the protein targets and functions for many of the F-box proteins and corresponding SCF complexes remain largely unknown in humans. However, the few well-characterized F-box proteins (e.g., SKP2, Cyclin F [CCNF], beta-transducin repeat containing E3 ubiquitin protein libase [βTrCP]) regulate substrates (e.g., cyclin E1 [13], cell division cycle 6 [CDC6] [14], and PLK4 [15]) involved in cellular processes that are essential for genome stability including cell cycle progression, centrosome duplication and dynamics, DNA replication and repair, and signal transduction and transcription. Thus, in-depth genetic and biochemical studies will be required to advance our rudimentary knowledge of each SCF complex and to reveal their specific protein targets and the biological processes they regulate. Gaining a greater understanding of each individual SCF complex component (e.g., SKP1, CUL1, RBX1, and the F-box proteins) is critical, as genetic alterations involving E3 subunits have detrimental impacts on the cell and are often implicated in disease pathophysiology (detailed in subsequent sections). For example, mutation, misexpression, or misregulation of the adaptor protein, SKP1, is expected to be especially damaging as it would prevent proper SCF complex formation, F-box protein recruitment, protein target poly-ubiquitination, and proteolytic degradation, which would adversely impact key biological pathways, culminating in cellular dysfunction and disease development. Indeed, *SKP1* copy number losses are suggested to be pathogenic driver events in colorectal and high-grade serous ovarian cancers [16,17]. Thus, understanding the role each member of the SCF complex in diverse cellular contexts will be critical to gain novel insight into disease pathogenesis, and may hold diagnostic and/or prognostic implications that will be valuable in the clinical management of diseases such as cancer [18,19].

## 4. The SCF Complex Regulates Transcription and Cancer-Associated Signaling Pathways

The SCF complex plays an important role in gene transcription and regulates numerous cancer-related cell signalling pathways. In this regard, specific SCF complexes, such as SCF^βTrCP^ and SCF^FBXO28^, mediate the levels and activities of many proto-oncogenic transcription factors including Snail [20], β-catenin [21], and MYC [22]. These transcription factors establish gene expression profiles that promote cancer-associated phenotypes including increased cellular proliferation, survival, and migration. When misregulated, these processes drive tumorigenesis and cancer metastasis through the epithelial to mesenchymal transition (EMT). For example, Snail is a transcriptional repressor of E-cadherin that is normally poly-ubiquitinated and targeted for degradation by SCF^βTrCP^ following phospho-activation by glycogen synthase kinase 3 (GSK3). Direct Snail de-phosphorylation by small c-terminal domain phosphatase (SCP) inhibits its interaction with SCF^βTrCP^, leading to increases in Snail levels [23]. Additionally, inhibition of GSK3 mediated by NFκB signalling prevents Snail phosphorylation, thereby preventing its degradation. NFκB signalling further supports Snail activity by inducing COP9 signalosome subunit 2 (COPS2)-mediated inhibition of SCF^βTrCP^ [24]. In each case, the disruption of SCF^βTrCP^-mediated Snail poly-ubiquitination and subsequent degradation leads to increased Snail levels that repress E-cadherin expression and drive EMT, cellular migration, and cancer metastasis [20,23,24]. Interestingly, however, the role of SCF^βTrCP^ is both complex and contradictory, as it also regulates IκBα degradation, a negative regulator of the NFκB signalling pathway [21].

Another substrate of SCF^βTrCP^ is β-catenin, a key component of the canonical WNT signalling pathway. Like Snail, β-catenin is also phospho-activated by GSK3 to enable SCF^βTrCP^-mediated poly-ubiquitination and degradation [21]. Mutations interfering with β-catenin phosphorylation or with its binding to βTrCP result in β-catenin accumulation and constitutive canonical WNT pathway activation. WNT signaling inhibits GSK3, further increasing β-catenin levels and driving target gene transcription, cellular proliferation, invasion, de-differentiation, and tumor development. Accordingly, β-catenin overexpression in human cancers, such as melanoma, correlate with disease progression [25]. As the SCF complex is responsible for regulation of oncogenic transcription factors, alterations involving core complex components (i.e., SKP1, CUL1, or RBX1) that disrupt SCF complex function can also contribute to the dysregulation of downstream substrates, such as β-catenin or Snail, and promote oncogenesis.

Interestingly, not all poly-ubiquitinated SCF substrates are targeted to the proteasome for degradation. Cepeda and colleagues [22] functionally characterized the F-box protein FBXO28, which acts as a transcriptional co-factor to positively regulate MYC target gene expression. FBXO28 is phosphorylated by CDK1 and cyclin-dependent kinases 1 (CDK1) and 2 (CDK2) in S and G2 phases of the cell cycle, which stabilizes FBXO28, promoting poly-ubiquitination of MYC by SCF^FBXO28^. However, rather than being targeted for degradation, poly-ubiquitinated MYC physically interacts with phosphorylated FBXO28 at the promoters of MYC target genes to recruit chromatin remodelling enzymes (e.g., histone acetyltransferase P300) and ultimately increase MYC-driven transcription. Hyper-activation of this pathway leads to overexpression of MYC target genes, increased proliferation, and neoplastic transformation [22,26]. Expectedly, FBXO28 is overexpressed in several tumor types, including breast, ovarian, testicular, and neuronal cancers, while immunohistochemical studies of tumor microarrays confirmed that phosphorylated FBXO28 was frequently overexpressed and associated with larger, more advanced tumors and poor survival in breast cancer patients [22]. Furthermore, the expression of an FBXO28 dominant negative mutant or silencing of wild-type FBXO28 hindered SCF^FBXO28^ ubiquitination of MYC and MYC-dependent transcription, transformation, and tumorigenesis [22]. It is interesting to note that additional SCF complexes, including SCF^FBXW7^ [27] and SCF^SKP2^ [28], also bind and poly-ubiquitinate MYC to effectively mediate MYC expression and transcriptional activity, respectively. This inherent redundancy may be an important mechanism that allows for highly regulated, fine-tuned control of critical cancer-related proteins, such as MYC. These few examples highlight the complexity of various SCF complexes in the regulation of cancer-associated proteins and underscore the need for additional studies elucidating the critical roles that individual SCF complexes may have in transcription and downstream target regulation. Only once this has been completed will we obtain a more holistic understanding of the SCF complex and its potential role in disease pathogenesis and pathophysiology.

## 5. SCF Complex Activity Is Essential to Mediate Cell Cycle Control and Prevent Tumorigenesis

The mammalian cell cycle is a multi-faceted process that is strictly regulated for accurate replication and segregation of genetic material and cellular division to ultimately ensure appropriate development, function, and survival of an organism. In response to mitogenic or anti-proliferative signals, the cell cycle is predominately controlled through the action of CDKs, which are positively or negatively regulated by activating cyclins or CDK inhibitors (CKIs), respectively [29]. The complex interplay between these components regulates the phosphorylation-dependent activation of protein targets and translates the growth signals received by the cell into an appropriate response. To regulate progression through the various cell cycle stages, the abundance of cyclins and CKIs is tightly controlled and oscillates in a spatio-temporal manner. For example, nuclear cyclin E-CDK2 promotes DNA synthesis, while cyclin B-CDK1 initiates mitotic entry [30]. As such, the expression levels of each complex are high during the appropriate cell cycle phases and are rapidly degraded by the UPS upon completion of their stage-specific tasks to ensure unidirectionality and stepwise progression through the cell cycle. Thus, the SCF complex is critical for cell cycle progression as it controls the timely degradation of cyclins and CKIs. In fact, it is the SCF complex in conjunction with the anaphase-promoting complex/cyclosome (APC/C), a distinct E3 ubiquitin ligase that regulates anaphase initiation and mitotic exit, that coordinate cell cycle progression.

### 5.1. The SCF Complex Is Critical for the G1 to S-Phase Transition

Although the SCF complex was initially believed to only regulate G1/S progression, it is now understood to have wide-ranging roles throughout the cell cycle and is active from late G1 to early M phase [30,31]. During G1, protein synthesis and cell growth are stimulated by SCF complex activity. For example, recruitment of pProgrammed cell death 4 (PDCD4) via the F-box protein βTrCP (FBXW1A), promotes SCF^βTrCP^-mediated poly-ubiquitination and proteasomal degradation of PDCD4. This prevents inhibition of the eukaryotic translation initiation factor (eIF4A) by PDCD4, enabling efficient protein translation and cell growth to occur [32]. In association with cyclin D1, CDK4 and CDK6 phosphorylate and inactivate the retinoblastoma protein family members pRB, p107 and p130, activating the E2F-related transcription factors [33]. In turn, E2F induces the expression of cyclins A, B, and, E during G1; however, these cyclin–CDK complexes are largely inhibited by CKIs (P21; P27; P57) to prevent premature DNA synthesis or initiation of mitosis at this stage [34,35,36,37]. APC/C further inhibits CDK1 and CDK2 activity by targeting the F-box protein SKP2, cyclins, and the CDK1-activating phosphatase cell division cycle 25A (CDC25A) [38]. In late G1, APC/C inhibition corresponds with increases in SKP2 expression, which achieves maximal abundance in S-phase and G2. SKP2 is now recruited to the SCF complex to promote CKI degradation, enhance CDK activity, and promote cell cycle progression. For example, SCF^SKP2^-mediated poly-ubiquitination and degradation of P27 activates the cyclin E-CDK2 kinase to initiate the G1/S transition [39,40]. Accordingly, reduced APC/C and CKI activity, increased cyclin and CDK levels, and activation of E2F transcription factor family members are all orchestrated by SCF complexes to mediate the transition from G1 to S phase [30].

### 5.2. The SCF Complex Functions within S-phase to Promote M-Phase Entry

Once a cell enters S-phase, cyclin D1 is rapidly degraded through the poly-ubiquitination and proteolytic degradation enabled by SCF^FBXO4^ [41]. The activity of the S-phase complexes cyclin E-CDK2 and cyclin A-CDK2 promote DNA synthesis and S-phase progression [42]. Additionally, cyclin A-CDK2 maintains APC/C inhibition to ensure cyclin stability, while the E2F transcription factors induce early mitotic inhibitor 1 (EMI1), also known as FBXO5, expression to ensure continued APC/C inhibition throughout S-phase and G2 [43,44]. CDK1 activity is kept in check by the SCF^βTrCP^-mediated degradation of CDC25A [45], and as the cell approaches G2, S-phase cyclin levels begin to decrease. Both non-phosphorylated cyclin E and phosphorylated cyclin E bound to CDK2 are targeted for degradation by SCF^SKP2^ and SCF^FBXW7^ complexes, respectively [46,47]. Upon completion of S-phase, ribonucleotide reductase family member 2 (RRM2) is phosphorylated by CDKs leading to SCF^CyclinF^-mediated degradation, which prevents superfluous deoxyribonucleotide production [48].

Throughout G2, the cell prepares for M-phase transition, which is regulated predominantly by cyclin B-CDK1. Nuclear cyclin B levels are maintained low prior to G2/M by SCF^NIPA^ (Non-imprinted in Prader–Willi/Angelman Syndrome) to prevent premature mitotic entry [49]. However, by late G2 SCF^βTrCP^, no longer targets CDC25A, allowing CDC25A phosphatase to counteract the WEE1 G2 checkpoint kinase phosphorylation-dependent inhibition of CDK1. SCF^βTrCP^ further promotes CDK1 activation by targeting WEE1 for degradation at this stage [45], while cyclin B-CDK1 phosphorylates NIPA, preventing SCF^NIPA^ formation, resulting in cyclin B accumulation that ultimately drives mitotic entry [50].

### 5.3. An Essential Role for the SCF Complex in Mitosis

From prophase to metaphase, cyclin B-CDK1 regulates mitotic spindle assembly, nuclear envelope breakdown, and chromosome condensation. To promote mitotic progression, cyclin B-CDK1 indirectly upregulates APC/C. Briefly, cyclin B-CDK1 phosphorylates the APC/C inhibitor EMI1 (an F-box protein), enabling SCF^βTrCP^-mediated EMI1 degradation, thereby increasing APC/C activity [45]. APC/C stimulates SKP2 degradation reducing SCF^SKP2^ activity, and increases the levels of CKIs such as P27 and P21 [36,40]. Additionally, APC/C targets mitotic cyclins A and B for degradation to dramatically reduce CDK1 activity [51]. CDK1 and 2 remain inactive until required for the next G1/S transition. Once the spindle assembly (mitotic) checkpoint is satisfied following bipolar attachment of paired sister chromatids that exhibit tension, the APC/C promotes securin degradation to initiate chromosome segregation (reviewed in [30,52]). Although APC/C is primarily responsible for anaphase onset, the SCF complex also contributes to this process as SCF^βTrCP^ targets the mitotic spindle assembly protein, BORA (Aurora Kinase A Activator) for degradation, allowing progression through anaphase and mitotic spindle disassembly [53]. Finally, the APC/C mediates chromosome decompaction, cytokinesis, nuclear envelope formation and subsequently re-establishes the G1 environment [30,54], when the entire cell cycle regulatory process can begin again.

### 5.4. Aberrant SCF Complex Expression and Function Adversely Impacts Cell Cycle Progression

The examples presented in the preceding sections illustrate how SCF complex-mediated degradation of positive and negative cell cycle regulators is essential to maintain both genome stability and cell cycle control. Mutation, misexpression, and/or misregulation of the individual SCF complex components are therefore predicted to induce cellular dysfunction, unchecked proliferation, and genome instability [55]. In fact, many of the genes encoding the F-box proteins presented above are frequently altered in cancer. For example, the βTrCP gene (*BTRC*) is somatically altered in various cancer types [56,57,58] and the aberrant protein exhibits both oncogenic and tumor suppressive activities within cells. βTrCP is frequently overexpressed in colorectal [59], pancreatic [60], and hepatoblastoma tumors [61], whereas loss-of-function alterations occur in gastric cancers [62]. Additionally, SKP2 (SCF^SKP2^), the F-box protein required for S-phase entry, is overexpressed in numerous cancers including breast [63], Kaposi’s sarcoma [64], T-cell lymphoma [65], and melanoma [66], with increasing *SKP2* expression correlating with diminished P27 (CKI) expression, advanced cancer progression, and poor patient prognosis [63,64,65,66]. Interestingly, siRNA-based silencing of *SKP2* in melanoma cells causes increases in P27 abundance and growth suppression, both in in vitro and in vivo mouse models [67], while *SKP2* knockout mice exhibit increases in cyclin E and P27 expression levels, polyploidy, supernumerary centrosomes, proliferation defects, and increases in apoptosis [13]. Collectively, these data suggest that, under normal conditions, βTrCP and SKP2 expression and function are tightly regulated to maintain accurate cell cycle progression, ensure genome stability, and prevent cancer development.

Certain F-box proteins predominately exhibit tumor-suppressive functions in cell cycle control. For example, FBXO4 is frequently under-expressed or somatically mutated in specific tumor types (e.g., esophageal carcinomas), which impairs SCF^FBXO4^ activity and contributes to cyclin D1 overexpression. Cyclin D1-dependent transcription of cyclins (detailed above) drives cell cycle progression and oncogenic transformation [41,68], as established by *FBXO4* knockout mice that develop lymphomas and dendritic cell sarcomas, along with mammary, uterine, and hepatocellular carcinomas [69]. Similarly, the F-box tumor suppressor *FBXW7* [70], exhibits loss of function alterations in ~6% of cancers [56,57,58] with inactivation preventing SCF^FBXW7^-dependent proteolytic degradation of cyclin E, which corresponds with increases in aneuploidy, nuclear aberrations, micronucleus formation, multi-polar spindles, as well as chromosome congression, cohesion, and segregation defects [71,72]. Cyclin F (FBXO1) is yet another F-box protein suspected to be a tumor suppressor as it is under-expressed in ~60% of hepatocellular carcinomas and reduced expression correlates with increased tumor sizes and numbers, advanced grade, stage and poor patient outcomes [73]. Conversely, F-box proteins can also harbor opposing oncogene-like activities. For example, EMI1 is a predicted oncoprotein that is frequently overexpressed in many cancers including lymphoma, ovarian, and hepatocellular carcinoma [74]. Conceptually, increased EMI1 expression prevents APC-mediated degradation of SKP2 and the mitotic cyclins, which will promote cell cycle progression [43,51,74]. In ovarian and hepatocellular carcinoma, EMI1 overexpression induces increases in cellular proliferation and tetraploidization, and is predictive of advanced tumor grade and poor prognosis [74]. Evidently, the SCF complexes regulating cell cycle progression can harbor tumor suppressor and/or oncogenic activities if not properly regulated in a spatio-temporal manner, which can lead to neoplastic transformation.

## 6. The Core SCF Complex Members Are Frequently Altered in Cancer

While genetic alterations or misregulation of the individual F-box proteins are associated with genomic instability and cancer pathogenesis, less is known about the tumorigenic roles of the invariable SCF components SKP1, CUL1, and RBX1. Conceptually, disruption of core SCF complex members is expected to produce complex outcomes involving the misregulation of a large number of protein targets, and may induce more severe or extensive aberrant phenotypes than those attributed to the misregulation of individual F-box proteins. To investigate this hypothesis, genetic and biochemical studies are required to fully realize the implications that SKP1, CUL1, and RBX1 dysfunction have for cell cycle misregulation, cellular dysfunction, genome instability, and tumorigenesis. Indeed, several genetic studies performed in malignant [17] and non-malignant [16] human contexts have established that reduced *SKP1*, *CUL1*, and *RBX1* expression induces chromosome instability (CIN), a prevalent form of genome instability. Briefly, CIN is defined as an increase in the rate at which whole chromosomes or large chromosome fragments are gained or lost, and is an established driver of both genetic and cellular heterogeneity (reviewed in [19,75]). Importantly, CIN is an enabling hallmark of cancer [55], associated with early disease events including cellular transformation [76,77] and intra-tumoral heterogeneity [78,79,80]) and late disease events such as metastasis [81,82,83,84], drug resistance [85,86], and poor patient survival (reviewed in [19]).

**Figure 3:**
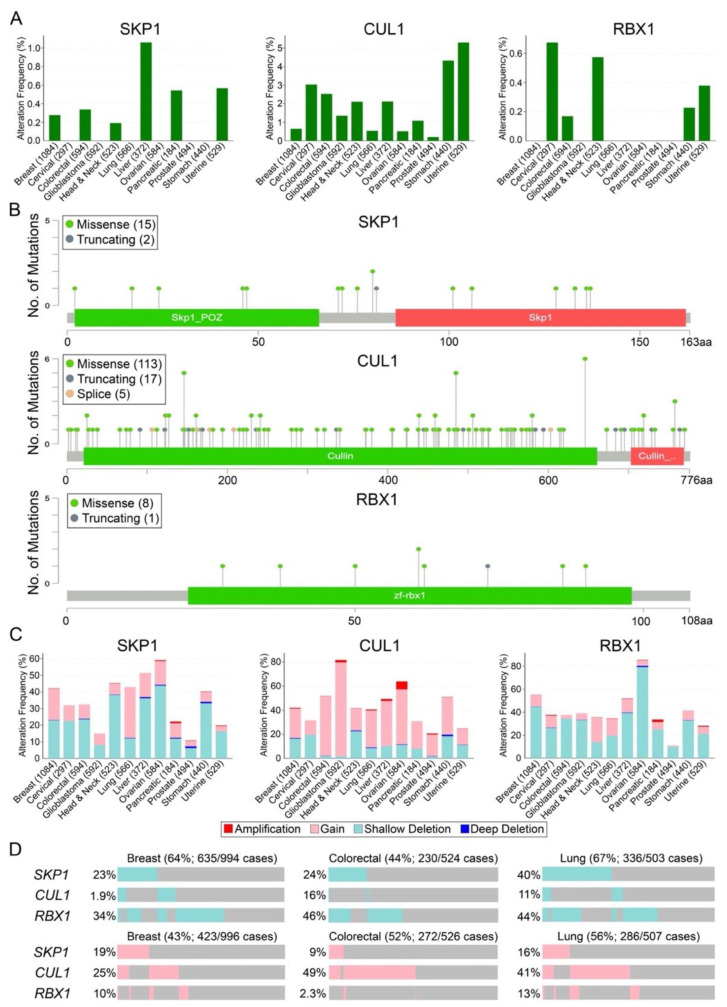
Mutation frequencies of the three core SCF complex members in 12 common cancer types. (**A**) The frequency of total *SKP1* (left), *CUL1* (middle) and *RBX1* (right) mutations, including missense, truncating, inframe, and fusions in 12 common cancer types (total cases) assessed using TCGA pan-cancer atlas data [58]. Note that missense and truncating mutations were identified for all three genes, while *CUL1* also harbors splice mutations. (**B**) Schematics from cBioPortal [56,57] mapping the protein positions of the corresponding mutations in *SKP1* (top), *CUL1* (middle), and *RBX1* (bottom). Note that, in general, the distribution of the encoded mutations is spread across the entirety of each protein in agreement with a tumor suppressor mutational load. (**C**) Prevalence of *SKP1* (left), *CUL1* (middle), and *RBX1* (right) copy number alterations including deep deletions, shallow deletions, gains, and amplifications in 12 common cancer types (total cases) [56,57,58]. Note that, in general, *SKP1* and *RBX1* exhibit more frequent copy number losses, whereas *CUL1* generally exhibits more gains. (**D**) Oncoprint data for breast, colorectal, and lung cancers depicting the individual and cumulative frequencies of *SKP1*, *CUL1*, and *RBX1* shallow deletions (top) or copy number gains (bottom) [58]. Vertical alignments within either the shallow deletions or gains categories identify samples from the same patient; patient-specific comparisons cannot be made between categories.

Patient-based data from The Cancer Genome Atlas (TCGA) provides further evidence supporting the possibility that aberrant expression and/or function of the core SCF complex components (*SKP1*, *CUL1*, and *RBX1*) have pathogenic roles in cancer [58]. Genetic analyses performed in cBioPortal (https://www.cbioportal.org, accessed on 2–12 May 2021) [56,57] reveal that the three members are somatically altered in 12 common cancer types (Figure 3A), and that non-synonymous mutations are rare, typically occurring in ≤1% of cancers for *SKP1* and *RBX1* or ≤5% for *CUL1* [58]. It should be noted however, that the *CUL1* coding sequence (NM_003592.3; 2331 base pairs [bp]; 776 amino acids [aa]) is 4.7- and 7.1-times larger than *SKP1* (NM_170679; 492 bp; 163 aa) and *RBX1* (NM_014248.4; 327 bp; 108 aa), respectively, and therefore the increased frequency of mutations may simply reflect its larger size. Nonetheless, the mutational loads are predominantly distributed across the entire coding sequence of each gene, supporting potential tumor suppressor functions (Figure 3B), rather than focally restricted to specific regions or hotspots typical of an oncogene [87,88].

Beyond somatic mutations, *SKP1*, *CUL1*, and *RBX1* gene copy number alterations including deep deletions (loss of 2 alleles), shallow deletions (loss of 1 allele), gains (gain of 1 allele), and amplifications (gain of ≥2 alleles) are frequently observed in all 12 cancer types (Figure 3C). In all instances, deep deletions and gene amplifications are extremely rare, generally occurring in <1% of all cancers, except for *CUL1* amplifications that occur in ~7% of ovarian cancer cases (Figure 3C). Furthermore, copy number losses occur most commonly for *SKP1* and *RBX1*, whereas copy number gains are most prevalent for *CUL1* [56,57,58]. These findings suggest that gene expression thresholds may exist whereby complete loss of expression may be lethal to a cell (i.e., essential genes), while extreme increases in expression may disrupt stoichiometries of core components to severely impact normal cell function/homeostasis and induce lethality. Collectively, *SKP1* copy number losses (shallow and deep deletions) range from 7% to 44% in prostate and ovarian cancers, respectively, while *CUL1* and *RBX1* losses range from 2% (glioblastoma) to 23% (head and neck) and 10% (prostate) to 80% (ovarian), respectively. Conversely, copy number gains (gains plus amplifications) for *SKP1*, *CUL1*, and *RBX1* range from 3% (uterine) to 31% (liver), 12% (cervical) to 80% (glioblastoma), and 1% (prostate) to 22% (head and neck), respectively.

Interestingly, when shallow deletions or gains of *SKP1*, *CUL1*, and *RBX1* are assessed concurrently within three commonly diagnosed cancers, there is a compounding effect (Figure 3D). More specifically, the combined frequencies of *SKP1*, *CUL1*, and *RBX1* shallow deletions are 64% (breast), 44% (colorectal) and 67% (lung), while the combined frequencies of *SKP1*, *CUL1*, and *RBX1* gains are 43% (breast), 52% (colorectal), and 56% (lung). Thus, while there are instances of individual patient tumors harboring simultaneous copy number alterations for more than one core SCF complex component (Figure 3D), this compounding effect suggests that many patients harbour only a single gene copy number alteration (i.e., mutual exclusivity). Considering that SKP1, CUL1, and RBX1 are core components contained within all SCF complexes, copy number alterations affecting these three genes have the potential to severely impair SCF complex expression and function, resulting in the misregulation of many substrates and cellular pathways. Intuitively, the proportion of cancers with aberrant SCF complex expression will increase when copy number alterations for the 69 F-box proteins are also considered. Collectively, these findings agree with the possibility that aberrant SCF complex expression and function may be a significant, yet underappreciated driver of many common cancer types. Importantly, while the copy number alterations detailed above suggest *SKP1*, *CUL1*, and *RBX1* may harbor tumor suppressor- and/or oncogene-like properties, these outwardly contrasting activities have also been described for many well-established cancer-associated genes including *BTRC* [56,57,58], *TP53* [89,90,91], *RAD54B* [92], and *USP22* [93].

**Figure 4:**
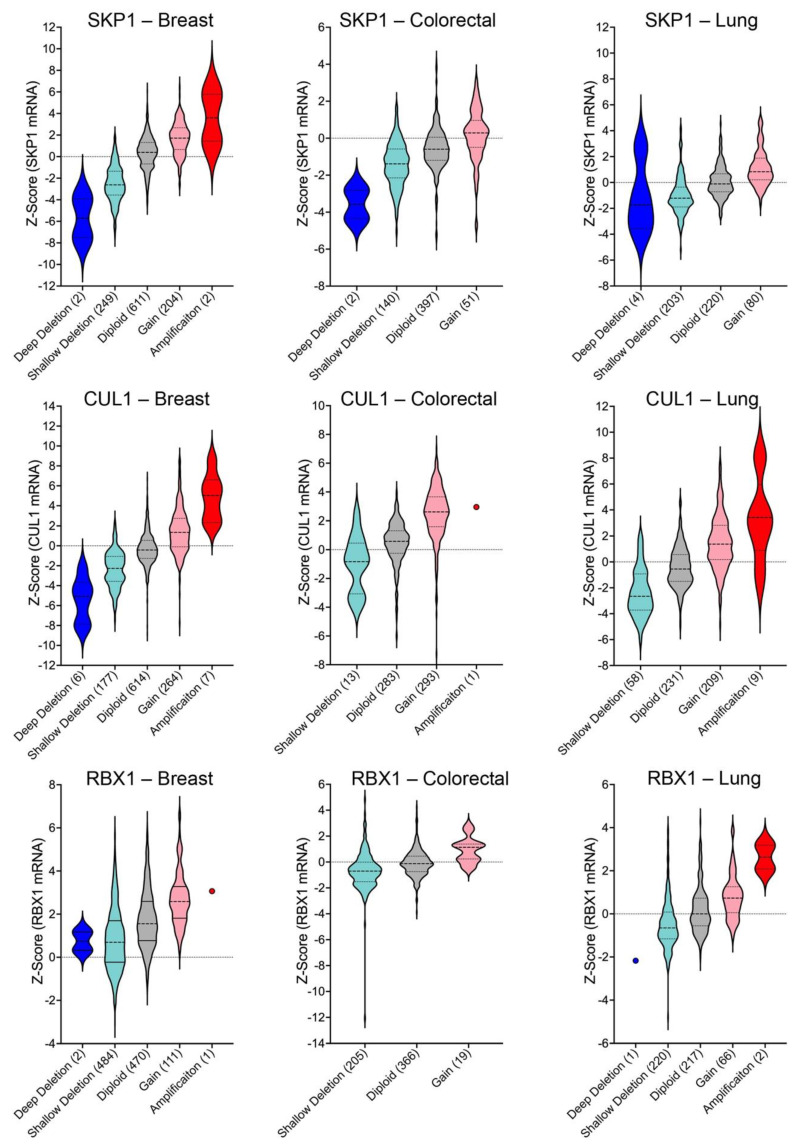
Gene copy number changes correspond with *SKP1*, *CUL1*, and *RBX1* expression levels. (A) Violin plots of TCGA pan-cancer data [58] from the three most commonly diagnosed cancers (i.e., breast, colorectal and lung). *SKP1* (**top**), *CUL1* (**middle**), and *RBX1* (**bottom**) copy number alterations (deep deletions; shallow deletions; gains; amplifications) and diploid cases are presented along the x-axis with total case numbers indicated within brackets. Note that categories with only single cases are identified by circles and that in general, deep deletions, and amplifications are rare.

**Figure 5:**
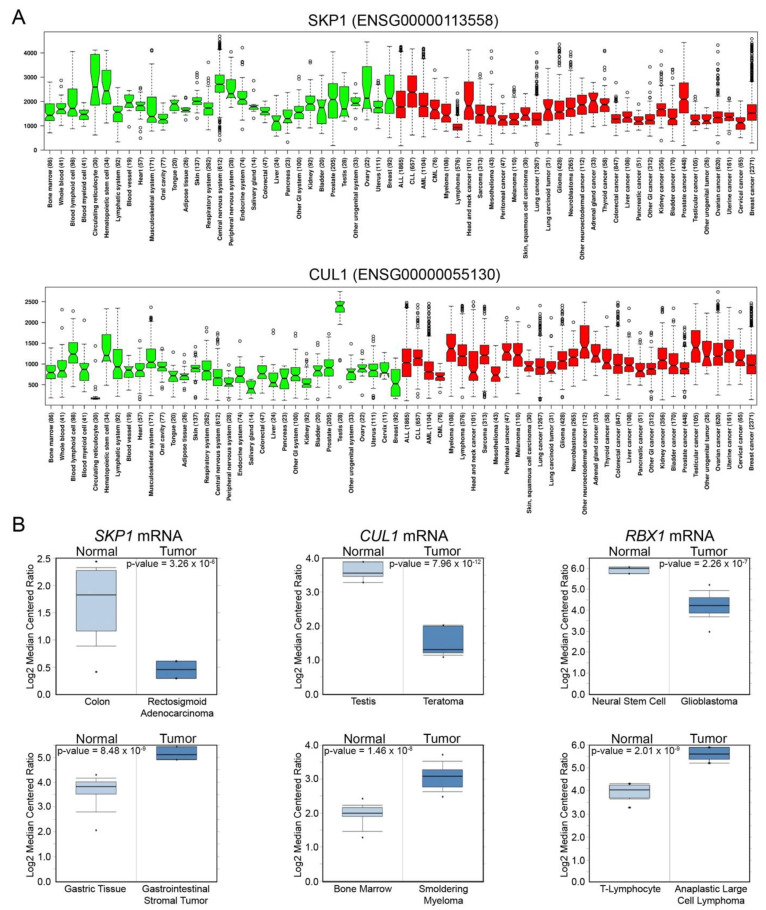
*SKP1* and *CUL1* exhibit altered expression in numerous cancer types. (**A**) Box-and-whisker plots displaying *SKP1* (**top**) and *CUL1* (**bottom**) mRNA expression levels in healthy (green) and cancer (red) tissues (total cases); *RBX1* expression data are not available. Boxes denote the interquartile range (25th, 50th, and 75th percentiles), while the whiskers extend 1.5-times the interquartile range and open circles identifying outliers. The notches in the boxes allow for visual comparisons between conditions; if two notches are non-overlapping, this is a strong indicator that the medians are different. Data and graph obtained using the in silico transcriptomics (IST) database [94]. In general, *SKP1* mRNA expression levels are frequently reduced in cancers relative to the corresponding normal tissues, whereas *CUL1* levels are predominantly increased. (**B**) Box plots displaying *SKP1*, *CUL1*, and *RBX1* mRNA expression levels for normal and tumor tissues. Boxes display the interquartile range with black dots identifying minimum/maximum values. Note that the top graphs display instances where significant decreases in mRNA expression occur within the tumors relative to normal tissue, while the bottom graphs provide examples where significant increases in expression occur. Graphs and statistical analyses were generated using the Oncomine database [95].

The above data suggest that copy number alterations for *SKP1*, *CUL1*, and/or *RBX1* adversely impact normal SCF complex function, resulting in the misregulation of many biological processes to ultimately drive cancer pathogenesis. Importantly, a central assumption behind this hypothesis is that the gene copy number alterations correspond with changes in expression at the level of the protein. Indeed, mRNA expression analyses of TGCA data [56,57,58] reveal robust and pervasive positive correlations between gene copy number alterations and gene expression levels. For example, in breast, colorectal, and lung cancers, increased copy numbers for *SKP1*, *CUL1*, and *RBX1* correspond with significant increases in mean expression values and ranges (Figure 4). The potential for both tumor suppressor-like and oncogene-like activities is further bolstered through analyses of additional gene expression datasets. In agreement with the predominant copy number alterations detailed above, mRNA expression analyses performed using the In Silico Transcriptomics (IST) database (https://ist.medisapiens.com, accessed on 2–12 May 2021) [94] reveal considerable variation in *SKP1* (ENSG00000113558) and *CUL1* (ENSG00000055130) mRNA expression in normal and tumor tissues (Figure 5A); unfortunately, no *RBX1* expression data are available. In agreement with the most prevalent copy number alterations detailed above, *SKP1* mRNA expression levels are often reduced within tumor samples relative to normal tissues, whereas *CUL1* expression is frequently increased. However, these relationships are not universal as some cancers do exhibit increases in *SKP1* expression relative to control tissues (e.g., chronic lymphocytic leukemia, head and neck, and liver). Likewise, several cancers, including chronic myelogenous leukemia and testicular cancer, exhibit decreases in *CUL1* expression. Moreover, supplementary mRNA expression data from the Oncomine database (https://www.oncomine.org, accessed on 2–12 May 2021) [95] corroborate that *SKP1, CUL1*, and *RBX1* can be significantly under or overexpressed within a variety of cancer types relative to control tissues (Figure 5B). Collectively, these findings support the possibility that *SKP1*, *CUL1*, and *RBX1* encode tumor suppressor- or oncogene-like activities depending on whether they are under- or over-expressed. In both cases, the altered expression of SCF complex components is expected to adversely impact SCF complex function and disrupt critical pathways required for genome stability, gene transcription, signal transduction and cell cycle progression, leading to cancer development and progression.

## 7. Targeting the SCF Complex for Cancer Treatments

Targeting the SCF complex, or more specifically the three invariable components (SKP1, CUL1, and RBX1), may appear counterintuitive due to the extensive array of protein targets and the multitude of biological pathways that the SCF complex regulates and the potential for adverse side effects. However, broad-spectrum approaches employing proteasome inhibitors, such as Bortezomib [96] or indirect SCF inhibitors, for example, MLN4924 (NEDD8-activating enzyme inhibitor that prevents CUL1 neddylation and indirectly inactivates CUL1) [97], have proven effective in the treatment of leukemia, lymphoma, and myeloma, lending support for the use of SCF complex inhibitors [98]. Additionally, pre-clinical and clinical evidence indicates that cancer cells harboring an aberrant UPS exhibit enhanced sensitivity to proteasome or SCF complex-targeting inhibitors relative to normal cells, thus enabling the administration of lower doses that still provide effective treatment with reduced overall side effects [99]. Based on these findings, therapeutic strategies exploiting aberrant SKP1, CUL1, or RBX1 expression and/or function represent promising avenues worthy of pursuit. Three distinct therapeutic strategies are presented below including synthetic genetic approaches, low-dose SCF inhibitors to exacerbate copy number loses of SCF complex members, and proteolysis-targeting chimeric molecules (PROTACS).

Concerted research efforts have recently focused on designing precision medicine approaches to therapeutically exploit the genetic aberrations driving cancer development and progression. In this regard, synthetic genetic approaches can be designed to target cancer cells harboring either copy number losses or gains. For example, the *SKP1*, *CUL1*, and *RBX1* copy number losses can be therapeutically exploited using synthetic lethal paradigms, analogous to how ovarian and breast cancers with reduced *BRCA1*/*2* expression are treated using poly [ADP-ribose] polymerase (PARP) inhibitors, such as olaparib or niraparib [100]. Briefly, synthetic lethality refers to a rare and lethal combination of two independently viable mutations (reviewed in [101]). Conceptually, a synthetic lethal strategy seeks to exploit the genetic defects contained within cancer cells (e.g., *SKP1*, *CUL1*, or *RBX1* loss) to induce lethality, while leaving the normal surrounding cells and tissues unaffected. Thus, the copy number losses and hypomorphic expression (loss-of-function) typically associated with tumor suppressor genes can be therapeutically exploited by downregulating or inhibiting a synthetic lethal interactor (drug target). Indeed, Burdova and colleagues [102] employed this strategy to uncover a synthetic lethal interaction between cyclin F (F-box protein) an CHEK1 inhibition, while Brough [103] et al. identified components of the SCF complex (e.g., *SKP1* and *SKP2*) as synthetic lethal interactors of *RB1* in triple negative breast cancer contexts. Similarly, copy number gains and hypermorphic expression (gain-of-function) associated with oncogenes could also be therapeutically exploited through a strategy referred to as synthetic dosage lethality (reviewed in [101]). First established in budding yeast [104], synthetic dosage lethal approaches are gaining in popularity due to the inherent challenges in developing drugs that target oncogenes without enzymatic activities, including transcription factors such as c-MYC. Thus, similar strategies could be employed to identify synthetic dosage lethal interactors (drug targets) for cells harboring copy number gains in *SKP1*, *CUL1*, or *RBX1*. As with synthetic lethal approaches, this systemic strategy would preferentially restrict killing to only those cancer cells that harbour increased expression of SCF complex components. In either case, synthetic genetic screening strategies using siRNA, shRNA, CRISPR/Cas9 techniques could be employed to identify candidate drug targets (synthetic lethal or synthetic dosage lethal interactors) capable of exploiting either copy number losses or gains. These approaches could consist of both direct tests, including candidate interactors identified through cross-species approaches, or unbiased approaches such as genome-wide screens (see [101]). Moreover, chemogenetic screens employing individual compounds or compound libraries could be extremely beneficial in identifying small molecule inhibitors that induce preferential killing of cancer cells with copy number losses or gains in *SKP1*, *CUL1*, or *RBX1*, which will have tremendous clinical utility across a myriad of cancer types. Alternatively, personalized strategies employing synergistic drug combinations targeting independent drug sensitivities may also have therapeutic potential [105]. Thus, it is conceivable that small molecule inhibitors targeting two or more independent synthetic lethal interactors of the SCF complex (i.e., those encoding functions within distinct biological pathways) may prove especially effective in combating cancers with copy number losses in SCF complex member genes. In any case, these synthetic lethal interactors, alone or in combination, will have to be evaluated for their clinical utility and pan-specificity across a myriad of cancer types and patient samples.

As an alternative strategy, low-dose SCF complex (SKP1, CUL1, or RBX1) inhibitors may prove beneficial in cancers already harboring shallow deletions (single allele loss). If, as predicted, all three genes are essential, then further diminishing or inhibiting their residual expression and/or function below a specific threshold is expected to induce lethality. Still, this hypothetical systemic approach is not without risk as each gene was recently identified as a CIN gene [16,17,106] and CIN is an enabling hallmark associated with early (e.g., cellular transformation [76,77] and intra-tumoral heterogeneity [78,79,80]) and late (e.g., metastasis [81,82,83] and drug resistance [85,86]) etiological events. Thus, such a systemic approach is expected to induce side effects within the normal surrounding cells or tissues; however, if reduced expression or inhibition can be selectively delivered to the cancer cells, then a large therapeutic window is expected to open. Nevertheless, rigorous genetic, biochemical and pharmacologic pre-clinical and clinical studies will be essential to discern whether this strategy will become a viable treatment option.

A final emerging therapeutic approach that may prove effective in cancers with *SKP1*, *CUL1*, or *RBX1* alterations is PROTACS (reviewed in [107,108,109,110]). Briefly, PROTACS are chimeric proteins that link a protein target to an F-box protein for SCF-mediated proteolytic degradation [109]. Conceivably, PROTACS could enable conditional or tissue-specific destruction of overexpressed proteins by enabling the targeted destruction of cancer cells harboring: (1) hypermorphic expression of SKP1, CUL1, or RBX1 due to copy number gains in those specific genes; or (2) the downstream proteins (i.e., oncogenes such as cyclin E1 [16,17]), normally targeted by the SCF complex for proteolytic degradation, but exhibit increased abundance due to *SKP1*, *CUL1*, or *RBX1* copy number losses. Although theoretical, several teams have already developed PROTACS targeting key oncogenes and ‘undruggable’ pathway genes (e.g., Myc [111,112]) found many different cancers (e.g., multiple myeloma, mantle cell lymphoma, and Burkitt’s lymphoma [113,114,115]). Accordingly, it may be feasible to create the chimeric molecules to target either of the contexts described above.

In summary, the therapeutic strategies detailed above mandate a deeper understanding of the aberrant genetics and biology that promote carcinogenesis to enable effective precision medicine approaches. Nevertheless, and before these strategies can come to fruition, additional studies are required to better characterize SKP1, CUL1, and RBX1, the associated 69 distinct SCF complexes with respect to the biological pathways that they regulate, and their individual impacts on cancer development and progression. Additionally, a myriad of future studies are required to identify candidate drug targets, test small molecule inhibitors, and optimize lead compounds before preclinical and ultimately clinical trials assessing drug safety and efficacy can be pursued. Thus, the translation of aberrant SCF complex biology into the clinic represents a critical new area of research that may offer tremendous promise for the development of novel and highly effective precision medicine strategies aimed at improving the lives and outcomes of those living with cancer.

## Data Availability

Patient-related data from Figure 3, Figure 4 and Figure 5A,B are based on data available from TCGA Research Network (https://www.cancer.gov/tcga) [58], IST [94] and Oncomine [95], respectively, and accessed online from 2–12 May 2021.
